# A retrospective risk factor analysis in psychiatric inpatients with COVID‐19 from 2020 to 2023 at a neuropsychiatric hospital in Tokyo

**DOI:** 10.1002/pcn5.70230

**Published:** 2025-11-05

**Authors:** Takuma Inagawa, Hajime Ariga, Hiroki Okano, Takako Enokida, Takashi Usami, Atsushi Unuma, Yuji Saitoh, Naoko Satake, Takamasa Noda

**Affiliations:** ^1^ Department of Psychiatry, National Center of Neurology and Psychiatry Hospital National Center of Neurology and Psychiatry Tokyo Japan; ^2^ Department of Gastroenterology, National Center of Neurology and Psychiatry Hospital National Center of Neurology and Psychiatry Tokyo Japan; ^3^ Home Medical Care Unit, Okada Clinic Tokyo Japan; ^4^ Department of Psychiatry Fukushima Medical Center of Mental Health Fukushima Japan; ^5^ Department of Behavioral Medicine, National Center of Neurology and Psychiatry National Institute of Mental Health Tokyo Japan; ^6^ Department of Bioresources, Medical Genome Center National Center of Neurology and Psychiatry Tokyo Japan; ^7^ Department of Psychiatry Fukuoka Prefectural Psychiatry Center Dazaifu Hospital Fukuoka Japan; ^8^ Department of Drug Dependence Research, National Center of Neurology and Psychiatry National Institute of Mental Health Tokyo Japan; ^9^ Department of Neurology, National Center of Neurology and Psychiatry Hospital National Center of Neurology and Psychiatry Tokyo Japan; ^10^ Department of Neurology Tokyo Metropolitan Neurological Hospital Tokyo Japan; ^11^ National Institute for Health Crisis Management Chiba Japan to National Institute for Health Crisis Management Chiba Japan; ^12^ Department of Community Mental Health and Law, National Center of Neurology and Psychiatry National Institute of Mental Health Tokyo Japan

**Keywords:** COVID‐19, National Center of Neurology and Psychiatry, neurology, pandemic, psychiatry

## Abstract

**Aim:**

To identify the factors associated with the severity and prognosis of coronavirus disease 2019 (COVID‐19) in patients with psychiatric or neurological disorders.

**Methods:**

We retrospectively analyzed the clinical characteristics and treatment outcomes of 349 patients with those disorders who were admitted for COVID‐19 at the National Center of Neurology and Psychiatry between June 2020 and March 2023. The study period was divided into three phases: June–December 2020, January–December 2021, and January 2022 to March 2023 according to the differences in types of COVID‐19 variants and our clinical care systems. Differences across phases were analyzed using the Kruskal–Wallis test. Logistic regression was performed to identify factors associated with moderate II or higher COVID‐19 and all‐cause mortality.

**Results:**

A total of 36 (10.3%), 107 (30.7%), and 206 (59.0%) patients were admitted in the first, second, and third phases, respectively. The average age of the patients was 66.4 ± 17.9 years, and 47% of them were female. Aspiration pneumonia complicated by COVID‐19 was strongly associated with moderate II or higher (odds ratio [OR] = 10.871, *p* < 0.001), antipsychotics (OR = 1.947, *p* = 0.034), transfer from psychiatric hospitals (OR = 2.878, *p* = 0.006), and older age (≥65 years, OR = 4.467, *p* < 0.001) were significant risk factors for moderate II or higher COVID‐19. Moderate II or higher COVID‐19 significantly predicted all‐cause mortality (OR = 32.658, *p* = 0.001). Phase‐stratified analyses indicated that older age (Phase 2: OR 3.69; Phase 3: OR 4.75) and aspiration pneumonia (Phase 3: OR 13.9) were consistently associated with moderate II or higher COVID‐19, whereas the association with antipsychotic use was phase‐dependent (significant in Phase 2: OR 4.74, but not in Phase 3: OR 1.12).

**Conclusion:**

These findings suggest that patients with psychiatric disorders, particularly elderly individuals or those with aspiration pneumonia, are associated with moderate II or higher COVID‐19. Further prospective observational studies are warranted.

## INTRODUCTION

The first confirmed case of COVID‐19 was reported in China in December 2019. The disease rapidly spread to other regions, and the World Health Organization declared the outbreak a global public health emergency on March 11, 2020. The first confirmed case of COVID‐19 in Japan was reported on January 15, 2020, that of Japanese on January 22, that in Tokyo on January 24, 2020, resulting in over 33 million COVID‐19 cases and more than 70,000 related deaths by March 2023 in Japan.^1^, ^2^, ^3^ On March 6, 2020, the first COVID‐19 patient with a psychiatric disorder was admitted to Tokyo Metropolitan Matsuzawa Hospital, Japan's largest psychiatric hospital.^4^ The first COVID‐19 cluster in a hospital in Tokyo was identified at Eiju General Hospital and that in a nursing care facility in March 2020.^5^, ^6^ As a result, the Japanese government began recommending or mandating hospitalization for patients with moderate or severe symptoms, the elderly, or pregnant women on April 2, 2020, and declared a state of emergency on April 7, 2020, urging Tokyo residents to refrain from nonessential outings, close their businesses, and cancel events.^7^, ^8^ As soon as a cluster of COVID‐19 infections, which involved four medical staff and five patients, was reported in a psychiatric hospital in Tokyo on May 28, 2020,^9^ the National Center of Neurology and Psychiatry (NCNP) established a COVID‐19 inpatient care unit with four beds designated for patients with comorbid COVID‐19 and psychiatric or neurological disorders on June 4, 2020, and the NCNP opened a dedicated COVID‐19 ward with 12–14 beds on February 10, 2021 because of rapid increase in hospitalizations due to COVID‐19. In this scenario, multidisciplinary collaboration was mandatory to manage both neuropsychiatric and physical symptoms, facilitate rehabilitation, and address patients' emotional well‐being during and after COVID‐19 infection. This collaboration was organized under our dedicated COVID‐19 treatment team (C‐team), which consisted of neurologists, psychiatrists, internists, neuropsychiatric nurses, pharmacists, social workers, psychologists, laboratory technicians, physical and occupational therapists, medical engineers, and administrative staffs from 2020 until 2023.

Providing adequate care for those patients during emergencies remains challenging in Japan, especially given the structural differences in psychiatric care between Japan and other countries. Japan has about 250 psychiatric beds per 100,000 people, which is much higher than the United Kingdom's 50 and the United States' 35.^10^ Additionally, around 70% of Japan's psychiatric beds are in specialized psychiatric hospitals, compared to only about 35% in the United States and a very small percentage in the United Kingdom. However, the treatment capacity in psychiatric wards in Japan is limited. For instance, university hospitals treat an average of 2.6 ± 6.8 patients with mental disorders and COVID‐19 annually, while general hospitals manage 3.5 ± 11.3 patients with mental disorders and COVID‐19 per year in 2022.^11^ Consequently, many psychiatric hospitals in Japan were not sufficiently equipped to manage patients during the COVID‐19 pandemic. Moreover, patients who presented with psychotic symptoms or agitation often had difficulty adhering to infection control measures, such as wearing masks or complying with quarantine protocols, thereby contributing to nosocomial transmissions. Additionally, the decreasing number of general hospitals with psychiatric beds made it more difficult for those patients to access specialized care.^12^ It is essential to develop a robust infectious disease response system for the management of those patients for future disease outbreaks or health emergencies. Previous studies indicated that a significant increase in the risk of COVID‐19 severity and mortality among patients with mental disorders,^13^, ^14^ and preexisting mental disorders, in particular, psychotic and mood disorders, and exposure to antipsychotics and anxiolytics were associated with COVID‐19 mortality.^15^ However, despite these studies, it remains unclear what kind of factors contributes to COVID‐19 severity and death in newly admitted COVID‐19 patients with psychiatric disorders in Japan. It is important to evaluate this aspect because Japanese psychiatric care systems were unique and quite different from those of other countries. Therefore, we aimed to analyze trends in COVID‐19 outbreaks, patient demographics, and clinical outcomes using data from COVID‐19 inpatient wards, and to identify the factors that are associated with the prognosis of COVID‐19 in patients with psychiatric disorders.

## METHODS

### Study design and participants

This was a single‐center, retrospective, chart review conducted at the NCNP Hospital. We followed the Strengthening the Reporting of Observational Studies in Epidemiology (STROBE) guidelines for observational studies (Supporting Information S2: Table 1).^16^ In our study, an observational study was performed by using the electronic medical records of all 349 patients admitted for COVID‐19 treatment between June 1, 2020, and March 31, 2023, at the NCNP. All consecutive COVID‐19 inpatients met our criteria, and there were no exclusion criteria applied. The sample size was not predetermined; 24‐h nursing care and ensured medication adherence through direct observation were provided. COVID‐19 diagnoses were confirmed using antigen tests or SARS‐CoV‐2 reverse transcriptase–polymerase chain reaction testing. The date of the first symptom onset or the date of a positive test (for asymptomatic patients) was defined as the onset of COVID‐19.

### Outcome measures and covariates

The patient data extracted from the medical records included age, sex, psychiatric or neurological diagnosis, comorbidities at admission (aspiration pneumonia,^17^ hypertension, diabetes, chronic kidney disease, chronic obstructive pulmonary disease, cardiovascular disease, and obesity), date of admission and discharge, source of admission, discharge destination, use of steroids and nasal high‐flow oxygen, COVID‐19 severity during hospitalization (after admission) psychotropic medications (types and doses), in‐hospital all‐cause mortality, and pandemic phase.

The study period was divided into three phases:

- Phase 1: June–December 2020 (specialized beds, conventional strains).
- Phase 2: January–December 2021 (specialized wards, Alpha and Delta strains).
- Phase 3: January 2022–March 2023 (specialized wards, Omicron strain).

Psychiatric diagnoses were classified according to the International Statistical Classification of Diseases and Related Health Problems, 10th revision (ICD‐10).^18^ COVID‐19 severity was categorized based on the clinical guidelines provided by the Japanese Ministry of Health, Labor and Welfare^7^:

- Mild: Peripheral oxygen saturation (SpO₂) > 96%, no respiratory symptoms or radiological evidence of pneumonia.
- Moderate I: SpO₂ 93%–96%, dyspnea or pneumonia findings, no oxygen required.
- Moderate II: SpO₂ < 93%, oxygen therapy required.
- Severe: Intensive care unit admission or mechanical ventilation, including nasal high‐flow.

COVID‐19 severity was assessed at admission based on the Japanese Ministry of Health guidelines, including oxygen saturation and respiratory support requirements. In patients with coexisting conditions such as aspiration pneumonia, severity classification still reflected COVID‐19–related respiratory compromise, consistent with clinical practice. Antipsychotic and benzodiazepine doses were converted to chlorpromazine equivalents (CP equivalent)^19^ and diazepam equivalents (BZ equivalent),^20^ respectively. To identify the prognostic factors for COVID‐19 severity during hospitalization, the following variables were selected as independent variables: psychiatric diagnosis (ICD‐10 F0–F9, others), admission source (psychiatric hospital, general hospital, facility, and home), vaccination status, CP‐equivalent dose, BZ‐equivalent dose, and comorbid aspiration pneumonia on admission. We also assessed the presence or absence of antipsychotic medications and benzodiazepines.

The primary dependent variable was moderate II or higher COVID‐19. All‐cause mortality during hospitalization was also analyzed as a dependent variable, with moderate II or severe set as an independent variable in logistic regression models.

### Statistical analysis

All statistical analyses were performed using spss Statistics version 24.0 and Microsoft Excel 365.

#### Trends in COVID‐19 outbreaks, patient demographics, and treatment outcomes

Differences in COVID‐19 outbreaks, patient demographics, and treatment outcomes between the three pandemic phases were evaluated using the Kruskal–Wallis test for nonparametric comparisons. Statistical significance was set at *p* < 0.05 (two‐tailed). The results are presented as percentages (for categorical variables) or means ± standard deviations (for continuous variables), along with *H*‐statistics and *p*‐values.

#### Prognostic factors for COVID‐19 severity and all‐cause mortality in patients with mental disorders

Logistic regression analysis was performed to identify factors associated with moderate II or higher COVID‐19 and all‐cause mortality in patients with psychiatric disorders. In our analysis, we defined mortality as death from any cause during hospitalization. The models created are described below:

- Model 1: Unadjusted binary logistic regression was performed to estimate odds ratios (ORs) and 95% confidence intervals (CIs) between independent variables and outcomes (moderate II or higher COVID‐19 and all‐cause mortality).
- Model 2: Adjusted for age, sex, and pandemic phase.
- Model 3: Further adjusted for comorbidities known to be associated with moderate II or higher COVID‐19 (diabetes, hypertension, chronic kidney disease [estimated glomerular filtration rate <60], chronic obstructive pulmonary disease, and obesity [body mass index (BMI) > 30]).

We set the pandemic phase (virus strain) as a covariate because the sample size was small and unbalanced among the three groups. To ensure sufficient statistical power in our risk factor analysis, we combined patient data across all three pandemic phases. Given the relatively small and unbalanced sample sizes in Phases 1–3, separate analyses for each phase would have lacked adequate power and precision. To account for potential systematic differences among phases—such as the dominant SARS‐CoV‐2 variant strains and changes in treatment protocols (e.g., corticosteroids, antivirals, and anticoagulants) over time—we included the variable “pandemic phase” as a covariate in all multivariable logistic regression models to account for systematic treatment differences across time. Actually, all patients received the standard of care based on the prevailing national or institutional guidelines at the time of admission, so these medications were generally prescribed in response to disease severity, and thus were not modeled directly as predictors to avoid introducing bias through overadjustment. Continuous variables such as age, CP equivalents, and BZ equivalents were binarized as follows: age, ≥65 versus <65 years; CP equivalents, ≥600 mg versus <600 mg; and BZ equivalents, ≥5 mg versus <5 mg.

These thresholds were based on clinical dosing guidelines.^19^, ^20^ A subgroup analysis was performed to identify factors that are associated with the outcomes of COVID‐19 treatment in patients transferred from psychiatric hospitals. Pearson correlation coefficients were calculated to analyze the time from symptom onset to hospitalization at our hospital as a surrogate marker for disease severity after admission.^21^, ^22^ We additionally conducted post hoc, phase‐stratified sensitivity analyses. For each pandemic phase (Phases 1–3), we re‐estimated the associations between aspiration pneumonia, antipsychotic use, and age ≥65 years and the outcome of moderate II or higher COVID‐19 using the same model specifications (Model 1: unadjusted; Model 2: adjusted for age and sex; and Model 3: additionally adjusted for comorbidities). Detailed results are provided in Supporting Information S2: Table 2.

## RESULTS

### Trends in COVID‐19 outbreaks, patient demographic characteristics, and treatment outcomes

**Figure 1:**
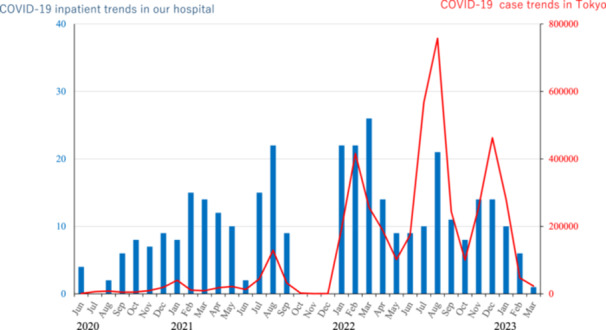
COVID‐19 ward inpatient trends.

A total of 349 patients were admitted to our hospital for COVID‐19 treatment during the study period: 36 (10.3%) in Phase 1, 107 (30.7%) in Phase 2, and 206 (59.0%) in Phase 3 (Table 1). Figure 1 illustrates the trends of COVID‐19 cases in Tokyo alongside inpatient trends in the hospital from 2020 to 2023. It highlights the temporal relationship between community infection waves and hospital admissions, reflecting how inpatient numbers fluctuated in response to pandemic surges over the 4‐year period. The mean age of the patients was 66.4 ± 17.9 years (70.2 ± 20.7 in Phase 1, 60.1 ± 17.3 in Phase 2, and 69.0 ± 17.0 in Phase 3 [*H* = 21.4, *p* < 0.01]). Regarding sex distribution, 168 (47%) of the patients were female.

**Table 1:** Trends in COVID‐19 outbreaks in different periods.

	Total	First period	Second period	Third period	Kruskal–Wallis
Number of patients	*n* = 349	*n* = 36	*n* = 107	*n* = 206	―
Age	66.4 ± 17.9	70.2 ± 20.7	60.1 ± 17.3	69.0 ± 17.0	*H* = 21.4, *p* < 0.01
Gender (M/F)	182/167	13/23	58/49	111/95	*H* = 2.77, *p* = 0.24
Admission days	18.4 (12.1)	14.2 (6.6)	21.9 (17.7)	17.4 (8.4)	*H* = 8.38, *p* = 0.02
Diagnosis (F0)	135 (38.6%)	18 (50.0%)	23 (21.5%)	94 (45.6%)	*H* = 8.27, *p* = 0.02
Diagnosis (F1)	13 (3.7%)	0 (0.0%)	9 (8.44%)	4 (1.94%)	*H* = 0.01, *p* = 0.99
Diagnosis (F2)	125 (35.8%)	9 (25%)	47 (43.9%)	69 (33.5%)	*H* = 1.90, *p* = 0.38
Diagnosis (F3)	22 (6.3%)	4 (11.1%)	6 (5.61%)	12 (5.83%)	*H* = 0.00, *p* = 0.99
Diagnosis (F4)	12 (3.4%)	1 (2.78%)	6 (5.61%)	5 (2.4%)	*H* = 0.01, *p* = 1.00
Diagnosis (F5–9)	32 (9.2%)	3 (8.33%)	13 (12.2%)	15 (7.28%)	*H* = 0.16, *p* = 0.99
Diagnosis (others)	3 (0.9%)	1 (2.78%)	3 (2.8%)	7 (3.4%)	*H* = 0.00, *p* = 1.00
Ad (MH)	259 (74.2%)	9 (25%)	78 (72.9%)	172 (83.5%)	*H* = 74.4, *p* < 0.01
Ad (GH)	10 (2.9%)	4 (11.1%)	4 (3.74%)	3 (1.46%)	*H* = 0.03, *p* = 1.00
Ad (nursing home)	31 (8.9%)	8 (22.2%)	5 (4.67%)	18 (8.73%)	*H* = 0.78, *p* = 0.96
Ad (homes)	48 (13.8%)	15 (41.7%)	20 (18.7%)	13 (6.31%)	*H* = 0.80, *p* = 0.67
Discharge (MH)	254 (72.8%)	11 (30.56%)	75 (70.1%)	168 (81.6%)	*H* = 7.17, *p* = 0.03
Discharge (GH)	12 (3.4%)	3 (8.33%)	7 (6.54%)	2 (0.97%)	*H* = 0.06, *p* = 1.00
Discharge (facilities)	29 (13.0%)	8 (22.2%)	5 (4.67%)	16 (7.77%)	*H* = 0.69, *p* = 0.97
Discharge (homes)	42 (12.0%)	14 (38.9%)	14 (13.1%)	14 (6.80%)	*H* = 0.68, *p* = 0.71
Death	12 (3.4%)	0 (0.0%)	6 (5.61%)	6 (2.91%)	*H* = 0.00, *p* = 1.00
Mild COVID‐19	211 (60.5%)	27 (75.0%)	51 (47.7%)	133 (64.6%)	*H* = 12.5, *p* < 0.01
Moderate I	48 (13.8%)	1 (2.78%)	23 (21.5%)	24 (11.7%)	*H* = 0.26, *p* = 0.88
Moderate II	61 (17.5%)	8 (22.2%)	14 (13.1%)	39 (18.9%)	*H* = 0.12, *p* = 0.94
Severe	29 (8.3%)	0 (0.0%)	19 (17.8%)	10 (4.85%)	*H* = 0.12, *p* = 0.94
Steroid use	56 (16.0%)	2 (5.56%)	39 (36.5%)	15 (7.28%)	*H* = 1.98, *p* = 0.37
Aspiration pneumonia	72 (20.6%)	1 (2.7%)	19 (17.8%)	52 (25.2%)	*H* = 0.85, *p* = 0.65
Vaccination coverage	149 (42.7%)	0 (0.0%)	2 (1.87%)	147 (71.4%)	*H* = 90.1, *p* < 0.01
Antipsychotics	246 (70.5%)	24 (66.7%)	80 (74.8%)	142 (68.9%)	*H* = 1.90, *p* = 0.39
Time from onset^a^	2.73 ± 2.17	3.35 ± 2.42	3.58 ± 2.79	2.18 ± 2.42	*H* = 13.8, *p* < 0.01
CP equivalent (Ad)	396.3 ± 619.9	219.8 ± 424.1	497.9 ± 601.2	374.4 ± 647.1	*H* = 7.94, *p* = 0.02
CP equivalent (Di)	411.3 ± 1007	200.5 ± 370.8	612.6 ± 1589	343.61 ± 590.1	*H* = 5.52, *p* = 0.06
Benzodiazepine	160 (45.8%)	18 (50%)	56 (52.3%)	86 (41.7%)	*H* = 2.34, *p* = 0.31
Diazepam eq (Ad)	6.45 ± 14.7	8.90 ± 22.8	7.49 ± 13.5	5.48 ± 13.3	*H* = 1.44, *p* = 0.49
Diazepam eq (Di)	5.13 ± 11.9	6.37 ± 15.7	5.91 ± 12.5	4.51 ± 10.6	*H* = 0.74, *p* = 0.69

*Note*: The average amount of medications is indicated as means (±standard deviation). F0, organic disorders; F1, mental and behavioral disorders due to psychoactive substance use; F2, schizophrenia, schizotypal, and delusional disorders; F3, mood disorders; F4, neurotic, stress‐related, and somatoform disorders; F5–9, other mental disorders, including behavioral syndromes associated with physiological disturbances and physical factors, disorders of adult personality and behavior, mental retardation, disorders of psychological development, and behavioral and emotional disorders with childhood and adolescence onset. CP equivalent and diazepam eq were presented as mg.

Abbreviations: Ad, admission; CP, chlorpromazine; Di, discharge; eq, equivalent; GH, general hospital; MH, mental hospital.

^a^ Mean days from COVID‐19 onset to hospitalization.

The overall mean length of hospitalization was 18.4 ± 12.2 days (14.2 ± 6.6 in Phase 1, 21.9 ± 17.7 in Phase 2, and 17.4 ± 8.4 in Phase 3 [*H* = 8.38, *p* = 0.02]).

The psychiatric diagnoses of the entire cohort were categorized as follows: F0 (organic disorders), *n* = 135 (38.7%); F1, *n* = 13 (3.7%); F2 (schizophrenia spectrum), *n* = 125 (35.8%); F3, *n* = 22 (6.3%); F4, *n* = 12 (3.4%); F5–9, *n* = 31 (8.9%); and others, *n* = 11 (3.2%). F0 diagnoses in Phase 3 were significantly low across the study phases: 50.0% (95% CI: 0–73.1) in Phase 1, 21.5% (95% CI: 0–38.3) in Phase 2, and 45.6% (95% CI: 0–55.7) in Phase 3 (*H* = 8.27, *p* = 0.02).

The admission routes included psychiatric hospitals (259 patients, 74.2%; 95% CI: 68.9–79.5), general hospitals (11 patients, 3.2%), institutions (31 patients, 8.9%), and private homes (48 patients, 13.7%). Admissions from psychiatric hospitals were significantly increased over time, with 25.0% recorded in Phase 1, 72.9% in Phase 2, and 83.5% in Phase 3 (*H* = 74.4, *p* < 0.01).

Regarding COVID‐19 severity, 211 patients had mild disease (60.5%; 95% CI: 53.9–67.1), 48 (13.8%) had moderate I disease, 61 (17.5%) had moderate II disease, and 29 (8.3%) had severe disease. The proportion of mild cases varied significantly across the study phases: 75.0% in Phase 1, 47.7% in Phase 2, and 64.6% in Phase 3 (*H* = 12.5, *p* < 0.01). Out of all the patients, 11 patients succumbed to death during their hospitalization. All of these cases were documented as deaths due to COVID‐19 on the death certificates, and one of these cases was reported as a clinicopathological case study.^23^

Vaccination coverage upon admission significantly increased after 2022 (0% in the first pandemic phase, 18.7% [2 patients] in the second, and 71.4% [147 patients] in the third [*H* = 90.1, *p* < 0.01]).

The proportion of patients whose primary diagnosis other than COVID‐19 (e.g., aspiration pneumonia or urinary tract infection) was 5.6% in the first phase, 10.3% in the second, and 23.3% in the third (*H* = 0.65, *p* = 0.72). Aspiration pneumonia was present in 2.8% of the patients in the first phase, in 17.7% in the second phase, and in 25.2% in the third phase (*H* = 0.85, *p* = 0.65). The number of patients who were taking antipsychotics on admission was 246 (70.5%), 24 (66.7%) in the first, 80 (74.8%) in the second, and 142 (68.9%) in the third (*H* = 1.90, *p* = 0.39). Mean CP equivalents on admissions were 396.3 ± 619.9 mg on admission, 219.8 ± 424.1 in the first, 497.9 ± 601.2 in the second, and 374.4 ± 647.1 in the third (*H* = 7.94, *p* = 0.02). On the other hand, the number of patients who were taking benzodiazepines was 160 (45.8%), 18 (50%) in the first, 56 (52.3%) in the second, and 86 (41.7%) in the third (*H* = 2.34, *p* = 0.31).

The trends in COVID‐19 outbreaks, patient demographic characteristics, and treatment outcomes are summarized in Table 1.

### Factors that are associated with the prognosis of COVID‐19 patients with psychiatric disorders

The results of logistic regression analysis of the association between variables and moderate II or higher COVID‐19 are presented in Table 2, whereas analysis of the association between the variables and death is summarized in Table 3.

**Table 2:** Logistic regression analysis of the association between variables and moderate II‐to‐severe COVID‐19.

Variables	Model 1		Model 2		Model 3	
	OR (95% CI)	*p*	OR (95% CI)	*p*	OR (95% CI)	*p*
F0	1.084 (0.660–1.779)	0.751	1.043 (0.615–1.767)	0.876	0.982 (0.550–1.753)	0.951
F1	1.928 (0.614–6.058)	0.261	1.875 (0.587–5.995)	0.289	0.502 (0.098–2.586)	0.410
F2	1.219 (0.739–2.011)	0.438	1.296 (0.776–2.166)	0.322	1.628 (0.951–2.786)	0.076
F3	1.134 (0.429–2.996)	0.799	1.112 (0.414–2.982)	0.834	0.609 (0.191–1.944)	0.402
F4	1.000 (0.265–3.780)	1	0.783 (0.192–3.191)	0.732	1.351 (0.332–5.496)	0.674
F5–9	0.551 (0.205–1.483)	0.238	0.564 (0.205–1.551)	0.268	0.962 (0.356–2.600)	0.939
Others	0.280 (0.035–2.217)	0.228	0.400 (0.048–3.358)	0.399	0.382 (0.043–3.433)	0.390
PH	2.414 (1.265–4.607)	0.008	2.658 (1.292–5.469)	0.008	2.878 (1.355–6.115)	0.006
GH	0.651 (0.138–3.073)	0.588	0.645 (0.130–3.215)	0.593	0.645 (0.127–3.272)	0.597
Facility	0.413 (0.140–1.214)	0.108	0.372 (0.124–1.117)	0.078	0.347 (0.109–1.099)	0.072
Home	0.477 (0.206–1.109)	0.085	0.518 (0.207–1.297)	0.16	0.474 (0.182–1.234)	0.126
Vaccine	0.714 (0.436–1.169)	0.181	0.625 (0.329–1.188)	0.152	0.572 (0.295–1.112)	0.100
Antipsychotics	1.954 (1.096–3.483)	0.023	2.124 (1.167–3.866)	0.014	1.947 (1.050–3.609)	0.034
CP eq	1.408 (0.819–2.419)	0.216	1.880 (1.049–3.368)	0.034	2.003 (1.091–3.679)	0.025
Benzodiazepine	0.927 (0.572–1.501)	0.757	1.175 (0.703–1.965)	0.538	1.182 (0.697–2.005)	0.535
Di eq	0.944 (0.576–1.547)	0.818	1.054 (0.627–1.772)	0.843	1.065 (0.625–1.814)	0.817
Aspiration	9.369 (5.252–16.714)	<0.001	9.951 (5.342–18.536)	<0.001	10.871 (5.690–20.771)	<0.001
Sex	0.641 (0.394–1.044)	0.074	0.609 (0.366–1.014)	0.057	0.600 (0.356–1.014)	0.056
Age	3.944 (2.205–7.054)	<0.001	4.121 (2.286–7.430)	<0.001	4.467 (2.351–8.489)	<0.001

*Note*: CP eq and diazepam equivalent were shown as mg.

Abbreviations: CI, confidence interval; CP, chlorpromazine; Di, discharge; eq, equivalent; GH, general hospital; OR, odds ratio; PH, psychiatric hospital.

**Table 3:** Logistic regression analysis of the association between diagnosis and COVID‐19‐related deaths.

Variables	Model 1		Model 2		Model 3	
	OR (95% CI)	*p*	OR (95% CI)	*p*	OR (95% CI)	*p*
F0	0.899 (0.258–3.130)	0.867	0.797 (0.227–2.801)	0.723	1.079 (0.277–4.197)	0.913
F1	‐	‐	‐	‐	‐	‐
F2	1.527 (0.456–5.107)	0.492	1.724 (0.500–5.942)	0.388	1.565 (0.435–5.636)	0.493
F3	1.505 (0.184–12.319)	0.703	1.645 (0.199–13.614)	0.644	1.837 (0.201–16.770)	0.590
F4	2.964 (0.348–2.964)	0.320	2.518 (0.212–29.866)	0.464	1.304 (0.078–21.824)	0.854
F5–9	‐	‐	‐	‐	‐	‐
Others	3.27 (0.381–28.066)	0.28	3.437 (0.388–30.467)	0.267	4.055 (0.424–38.780)	
PH	0.928 (0.241–3.577)	0.914	0.772 (0.179–3.338)	0.729	0.984 (0.207–4.764)	0.984
GH	3.270 (0.381–28.066)	0.28	3.651 (0.387–34.441)	0.258	3.348 (0.349–32.095)	0.295
Facility	1.023 (0.127–8.270)	0.983	1.033 (0.126–8.468)	0.976	1.883 (0.207–17.149)	0.574
Home	0.633 (0.079–5.059)	0.666	0.775 (0.089–6.790)	0.818	0.298 (0.024–3.752)	0.349
Vaccine	0.757 (0.217–2.634)	0.661	0.593 (0.133–2.648)	0.494	0.679 (0.137–3.357)	0.635
CP	1.866 (0.533–6.540)	0.33	2.195 (0.606–7.945)	0.231	1.953 (0.516–7.393)	0.324
Di	0.153 (0.019–1.206)	0.075	0.137 (0.015–1.263)	0.079	0.096 (0.008–1.153)	0.065
Aspiration	1.457 (0.376–5.635)	0.586	1.374 (0.346–5.459)	0.652	1.702 (0.402–7.206)	0.470
Moderate II/severe	32.658 (4.117–259.075)	0.001	47.538 (8.555–264.167)	<0.001	48.384 (5.211–449.254)	0.001
Sex	1.312 (0.393–4.381)	0.659	1.351 (0.397–4.595)	0.630	1.173 (0.326–4.216)	0.807
Age	7.020 (0.888–55.469)	0.065	6.995 (0.883–55.408)	0.065	‐	‐

*Note*: CP eq and diazepam equivalent were shown as mg. When the parameters to be estimated/analyzed cannot be identified from the datasets, the results were shown as “‐.”

Abbreviations: CI, confidence interval; CP, chlorpromazine; Di, discharge; GH, general hospital; OR, odds ratio; PH, psychiatric hospital.

Aspiration pneumonia was consistently associated with moderate II or higher COVID‐19 across all models:

- Model 1: OR = 9.369 (95% CI: 5.252–16.714), *p* < 0.001
- Model 2: OR = 9.951 (95% CI: 5.342–18.536), *p* < 0.001
- Model 3: OR = 10.871 (95% CI: 5.690–20.771), *p* < 0.001

Antipsychotics were significantly associated with moderate II or higher COVID‐19:

- Model 1: OR = 1.954 (95% CI: 1.096–3.483), *p* = 0.023
- Model 2: OR = 2.124 (95% CI: 1.167–3.866), *p* = 0.014
- Model 3: OR = 1.947 (95% CI: 1.050–3.609), *p* = 0.034

CP‐equivalent doses ≥600 mg were also significantly associated with moderate II or higher COVID‐19:

- Model 2: OR = 1.880 (95% CI: 1.049–3.368), *p* = 0.034
- Model 3: OR = 2.003 (95% CI: 1.091–3.679), *p* = 0.025

Admission from psychiatric hospitals was an independent predictor of moderate II or higher COVID‐19:

- Model 1: OR = 2.414 (95% CI: 1.265–4.607), *p* = 0.008
- Model 2: OR = 2.658 (95% CI: 1.292–5.469), *p* = 0.008
- Model 3: OR = 2.878 (95% CI: 1.355–6.115), *p* = 0.006

Older age (≥65 years) was a strong predictor of moderate II or higher COVID‐19 across all models:

- Model 1: OR = 3.944 (95% CI: 2.205–7.054), *p* < 0.001
- Model 2: OR = 4.121 (95% CI: 2.286–7.430), *p* < 0.001
- Model 3: OR = 4.467 (95% CI: 2.351–8.489), *p* < 0.001

Regarding all‐cause mortality, moderate II or higher COVID‐19 significantly predicted death across all models:

- Model 1: OR = 32.658 (95% CI: 4.117–259.075), *p* = 0.001
- Model 2: OR = 47.538 (95% CI: 8.555–264.167), *p* < 0.001
- Model 3: OR = 48.384 (95% CI: 5.211–449.254), *p* = 0.001

In post hoc, phase‐stratified sensitivity analyses (Supporting Information S2: Table 2), older age was associated with moderate II or higher COVID‐19 in Phase 2 (OR 3.69, 95% CI 1.01–13.54) and Phase 3 (OR 4.75, 95% CI 1.80–12.50), whereas the estimate in Phase 1 was imprecise (OR 1.38, 95% CI 0.13–14.40). Aspiration pneumonia showed strong associations in Phase 3 (OR 13.90, 95% CI 5.71–33.84) and a borderline effect in Phase 2 (OR 3.21, 95% CI 0.98–10.52), but could not be reliably estimated in Phase 1 due to sparse data. In contrast, the association with antipsychotic use was phase‐dependent, being significant in Phase 2 (OR 4.74, 95% CI 1.13–19.83) but not in Phase 3 (OR 1.12, 95% CI 0.52–2.44). These results are illustrated in Supporting Information S1: Figure 1.

## DISCUSSION

In this study, we analyzed 349 patients with psychiatric or neurological disorders across three pandemic phases. Approximately 74.2% of the patients were transferred to our hospital from psychiatric hospitals, and this proportion tended to increase over time. Our analyses suggested that older age, aspiration pneumonia, transfer from psychiatric hospitals, and higher doses of antipsychotic medications were associated with moderate II or higher COVID‐19, which significantly predicted all‐cause mortality. All of the 11 cases, who succumbed to death during their hospitalization, were documented as deaths due to COVID‐19. This study highlights the need for early intervention, vaccination prioritization, and specialized care for psychiatric patients during pandemics.

Older age remained a strong predictor of moderate II or higher COVID‐19, even after adjusting for confounding factors. Interestingly, the average patient age was significantly lower during the second wave of the pandemic. This may be due to the high transmissibility and severity of the Delta SARS‐CoV‐2 variant,^24^, ^25^ which was widespread during that period and rapidly spread among younger individuals, particularly in workplaces and schools. In contrast, older adults tend to experience more severe illness due to age‐related decline in immune function, which hampers viral clearance, and a higher risk of cytokine storms that can lead to acute respiratory distress syndrome.

The duration of hospitalization was longest in the second wave, with a mean hospitalization duration of 21.9 ± 17.7 days compared to the 17.4 ± 8.4 days recorded in the third wave (*p* = 0.02). This coincided with a reduced proportion of mild COVID‐19 cases (47.7%, *p* < 0.01). The Delta variant, which dominated during the second wave, was not only more contagious but also more severe illness.^26^, ^27^ COVID‐19 vaccine coverage was still limited at that time, as Japan began its vaccination program on February 17, 2021. By 2022, however, coverage among hospitalized patients had risen to 71.4% (*p* < 0.01), contributing to milder cases and shorter hospital stays. Timely vaccination rollout can reduce disease severity and hospital burden, highlighting the importance of early and widespread vaccine distribution during future outbreaks.

The proportion of patients diagnosed with organic mental disorders (F0) significantly reduced in Phase 2 (21.5%, *p* = 0.02), whereas CP‐equivalent antipsychotic doses significantly increased (497.9 ± 601.2 mg, *p* = 0.02). These findings suggest that during the Delta wave, more patients with schizophrenia, which requires higher doses of antipsychotics, were hospitalized. Due to the complexity of their conditions, such patients may have had limited access to care in other facilities, especially during periods of bed shortages.^28^ In addition, COVID‐19 clusters in psychiatric hospitals complicated the assessment and management of these patients.^29^ Consequently, patients with schizophrenia were more likely to deteriorate after admission.^30^, ^31^ Furthermore, antipsychotic doses were significantly associated with moderate II or higher disease, even after adjusting for confounders (OR = 1.947 [95% CI: 1.050–3.609], *p* = 0.034), and high dose antipsychotics (CP‐equivalent doses ≥ 600 mg) showed similar associations (OR = 2.003 [95% CI: 1.091–3.679], *p* = 0.025). These results were in line with the previous study that preexisting mental disorders and exposure to antipsychotics were associated with moderate II or higher COVID‐19.^15^ This study's findings did not imply that antipsychotic medications should be discontinued immediately in patients with COVID‐19. Actually, recent evidence suggests that in patients with chronic schizophrenia, even during hospitalization, indiscriminate reduction or discontinuation of antipsychotic medication may increase not only the risk of relapse but also the risk of mortality, particularly from suicide and cardiovascular causes.^32^ On the other hand, continued administration at an appropriate maintenance dose has been associated with a reduction in all‐cause mortality, with especially significant reductions reported at moderate dose levels.^33^ However, in patients with dementia, reducing or discontinuing antipsychotic medications during hospitalization may help lower the risk of all‐cause mortality.^34^ For individuals in the chronic phase of dementia, avoiding unnecessary long‐term use and considering dose reduction or discontinuation when possible—while carefully monitoring the patient—may be beneficial in improving survival rates and preventing cardiovascular complications.^35^ While antipsychotic use has been linked to moderate II or higher COVID‐19, treatment decisions should be individualized, as continued use may reduce mortality in chronic schizophrenia, whereas careful reduction or discontinuation may benefit patients with dementia. Future prospective studies should aim to improve integrated medical and psychiatric care systems, especially during public health emergencies.

Aspiration pneumonia was significantly associated with moderate II or higher disease, even after adjusting for confounders (OR = 10.871, *p* < 0.001). Patients with psychiatric disorders are more prone to swallowing difficulties, which are linked to the risk of aspiration.^36^ In particular, schizophrenia is linked to a 1.37‐fold higher risk of acute respiratory failure and a 1.34‐fold higher risk of needing mechanical ventilation.^37^ Some antipsychotic medications may further impair the swallowing reflex.^38^ Aspiration pneumonia can also worsen COVID‐19 by intensifying the immune response and potentially triggering cytokine storms. While steroids are used to control these storms, they can weaken the immune system and further aggravate aspiration pneumonia.^39^ In this study, higher CP‐equivalent antipsychotic doses were associated with moderate II or higher COVID‐19 (OR = 2.003, *p* = 0.025), possibly due to their immunosuppressive properties and negative impact on swallowing function.^40^ These findings suggested the need for careful monitoring of swallowing function and antipsychotic dosing in psychiatric patients to prevent aspiration pneumonia and reduce the risk of moderate II or higher COVID‐19.

Transfers from psychiatric hospitals peaked in Phase 3 (83.5%, *p* < 0.01), coinciding with the emergence of the highly transmissible Omicron variant.^41^ Psychiatric hospitals are especially vulnerable to outbreaks due to close living arrangements. During the first wave, hospitalizations were mostly linked to community transmission and spanned all age groups.^42^ Although shifts in variant prevalence did not drastically change overall transmission patterns, the reduced severity of COVID‐19 led to more frequent in‐hospital transmissions within psychiatric and nursing facilities.^43^, ^44^ In addition, our data showed that the average number of days from symptom onset to hospital admission significantly decreased across the three phases—from 3.35 days in Phase 1 to 2.18 days in Phase 3 (*H* = 13.8, *p* < 0.01). This trend likely reflects improvements in public health responses, including increased testing availability, greater community awareness, and more efficient referral systems for psychiatric patients. Earlier recognition of infection and faster linkage to care may have contributed to reducing the risk of delayed hospitalization and subsequent disease progression. Transfer from psychiatric hospitals was significantly associated with moderate II or higher COVID‐19 (OR = 2.878, *p* = 0.006), possibly due to unrecognized exposure and delayed treatment.^45^ Patients with psychiatric disorders also face higher risks of severe illness and mortality.^46^ However, as Japan's psychiatric care system differs markedly from those of other countries,^12^ we conducted a subgroup analysis to identify factors that were associated with the duration from symptom onset to hospitalization. We found that vaccination coverage was negatively correlated with this duration, suggesting that the vaccinated individuals received improved access to care and earlier intervention (Supporting Information S2: Table 3). Supporting Information S1: Figure 2 also shows monthly trends in COVID‐19 inpatient admissions at our hospital (blue bars), the average duration from symptom onset to hospitalization (green line), and overall COVID‐19 case trends in Tokyo (red line) from June 2020 to March 2023. The data illustrate that hospitalization trends in our facility did not always align with city‐wide case surges, and the duration from onset to hospitalization varied over time, decreasing gradually after 2021. Transfers from psychiatric hospitals were associated with moderate II or higher COVID‐19 cases, likely due to delayed detection and care, while increased vaccination coverage correlated with earlier hospitalization.　Enhancing vaccination programs and care pathways for institutionalized patients may help reduce moderate II or higher COVID‐19 outcomes and lessen healthcare strain. These findings also call for further investigation into the biological and systemic mechanisms linking psychiatric conditions and COVID‐19 severity. As a sensitivity check for temporal heterogeneity, phase‐stratified analyses yielded various patterns. In Phase 2, age ≥65 years remained significantly associated with moderate II or higher disease across all models, antipsychotic use showed a robust positive association, and the effect of aspiration pneumonia was strong in unadjusted models but attenuated to borderline significance after multivariable adjustment. In Phase 3, both age ≥65 years and aspiration pneumonia were consistently significant, whereas antipsychotic use was not. In Phase 1, estimates were imprecise due to a small sample size and complete separation for aspiration pneumonia. The observed difference in the association between antipsychotic use and COVID‐19 severity between Phase 2 and Phase 3 may be attributable to several factors including variations in patient profiles^14^, ^15^ and changes in COVID‐19 treatment strategies^47^ over time. Taken together, these analyses suggest that older age and aspiration pneumonia were relatively stable predictors across phases, whereas the influence of antipsychotic use on COVID‐19 severity appeared to be context‐specific and phase‐dependent. This highlights the importance of continuously reassessing risk factors as pandemic conditions, patient populations, and treatment strategies evolve.

This study has several notable strengths. It presents one of the largest single‐center datasets of COVID‐19 hospitalizations among patients with psychiatric or neurological disorders in Tokyo, covering three distinct phases of the pandemic. It also provides valuable insights into temporal trends and identifies key risk factors for moderate II or higher COVID‐19 and all‐cause mortality—such as aspiration pneumonia, high‐dose antipsychotics, older age, and admission pathways. Moreover, the use of appropriate statistical adjustments and adherence to the STROBE guidelines enhanced the reliability of the results.

However, this study also has several limitations. First, the overall sample size was relatively small. Second, the single‐center design limits the generalizability of the findings, particularly given the unique features of Japan's psychiatric care system. Third, the retrospective nature of the study restricts our ability to assess disease progression over time and may introduce recall bias. Our findings suggest a potential link between several factors and moderate or higher COVID‐19, though causality cannot be inferred from this study. Fourth, excluding nonhospitalized patients likely led to underrepresentation of milder cases. Fifth, institutional practices, resources, and patient demographics at the NCNP may differ from other facilities, affecting external validity. Sixth, we combined all patient data across three pandemic phases as a covariate to account for different dominant viral strains and evolving treatment protocols (the use of steroids, antivirals, and anticoagulants). Although we adjusted for “pandemic phase” in our multivariable models to account for these temporal differences, we could not fully exclude unmeasured confounding related to treatment variability and changes in clinical practice over time. Seventh, coexisting infections such as aspiration pneumonia at the time of COVID‐19 diagnosis, which may have contributed to respiratory compromise and thus influenced severity classification. Nevertheless, such coexisting conditions are commonly observed in psychiatric inpatients with COVID‐19, and their inclusion reflects the complexity of real‐world practice. Future prospective studies should further explore the interaction between co‐infections and COVID‐19 severity. Eighth, although we added post hoc phase‐stratified models in response to reviewer feedback, the Phase 1 sample was small and exhibited complete separation for aspiration pneumonia. While the direction of effects was generally consistent with the pooled results—particularly for age and aspiration pneumonia—phase‐specific estimates should be interpreted cautiously due to limited power and potential residual confounding unique to each period. Ninth, the swallowing function was not assessed in our dataset. Swallowing impairment, common among psychiatric populations, may mediate the relationship between age, antipsychotic use, aspiration pneumonia, and COVID‐19 severity, and thus represents an important unmeasured confounder. The tenth limitation is that while our study was conducted at a specialized national center with extensive resources and a multidisciplinary team, it is important to note that these findings may not be directly applicable to all psychiatric hospitals in Japan. The infrastructure and resources available at NCNP, including a dedicated C‐team, may not be present in all facilities. Lastly, clinical diagnoses and patient records were based on subjective judgment without the use of standardized assessment tools, which may have introduced inter‐rater bias.

## CONCLUSIONS

This study highlights the vulnerability of patients with psychiatric or neurological disorders to moderate II or higher COVID‐19, especially those with aspiration pneumonia, those who use high‐dose antipsychotics, and those who are transferred from psychiatric hospitals against emerging respiratory infectious diseases. In addition, this study demonstrated that older age and moderate II or higher COVID‐19 are strong predictors of all‐cause mortality in patients with psychiatric or neurological disorders. Notably, while older age and aspiration pneumonia tended to remain risk factors across different pandemic phases, the impact of antipsychotic use was not consistent and appeared to vary between phases. Policy efforts should enhance infection control and early detection in psychiatric facilities, where delays in hospitalization and aspiration are associated with moderate II or higher COVID‐19. Our findings underscore the urgent need for integrated medical and psychiatric care, early intervention, and infection control preparedness in mental health facilities. Further prospective observational studies are warranted.

## AUTHOR CONTRIBUTIONS

Takuma Inagawa and Takamasa Noda conceptualized this study and reviewed and advised on research plans. Takuma Inagawa conducted statistical analyses. Takuma Inagawa wrote the first draft of this manuscript. All the authors were involved in COVID‐19 care. All the authors read and approved the final draft of the manuscript.

## CONFLICT OF INTEREST STATEMENT

The authors declare no conflicts of interest.

## ETHICS APPROVAL STATEMENT

This study was approved by the Ethics Committee of the National Center of Neurology and Psychiatry (A2023‐028).

## PATIENT CONSENT STATEMENT

An opt‐out approach was adopted in this study for the acquisition of patient consent. The approach involved providing participants with the opportunity to decline having their results posted on the website of the Ethics Committee of the National Center of Neurology and Psychiatry.

## CLINICAL TRIAL REGISTRATION

Not applicable.

## Supporting information


**Supporting Information**.

**Supporting Information**.

## Data Availability

The data that support the findings of this study are available from the corresponding author upon reasonable request.
