# Supplementary material for: A retrospective risk factor analysis in psychiatric inpatients with COVID‐19 from 2020 to 2023 at a neuropsychiatric hospital in Tokyo
Source: PCN Rep. 2025 Nov 5;4(4):e70230. doi: 10.1002/pcn5.70230 (PMC12589898; doi:10.1002/pcn5.70230)

Supplementary Figure 1. Phase-Specific Adjusted Odds Ratios for Key Risk Factors

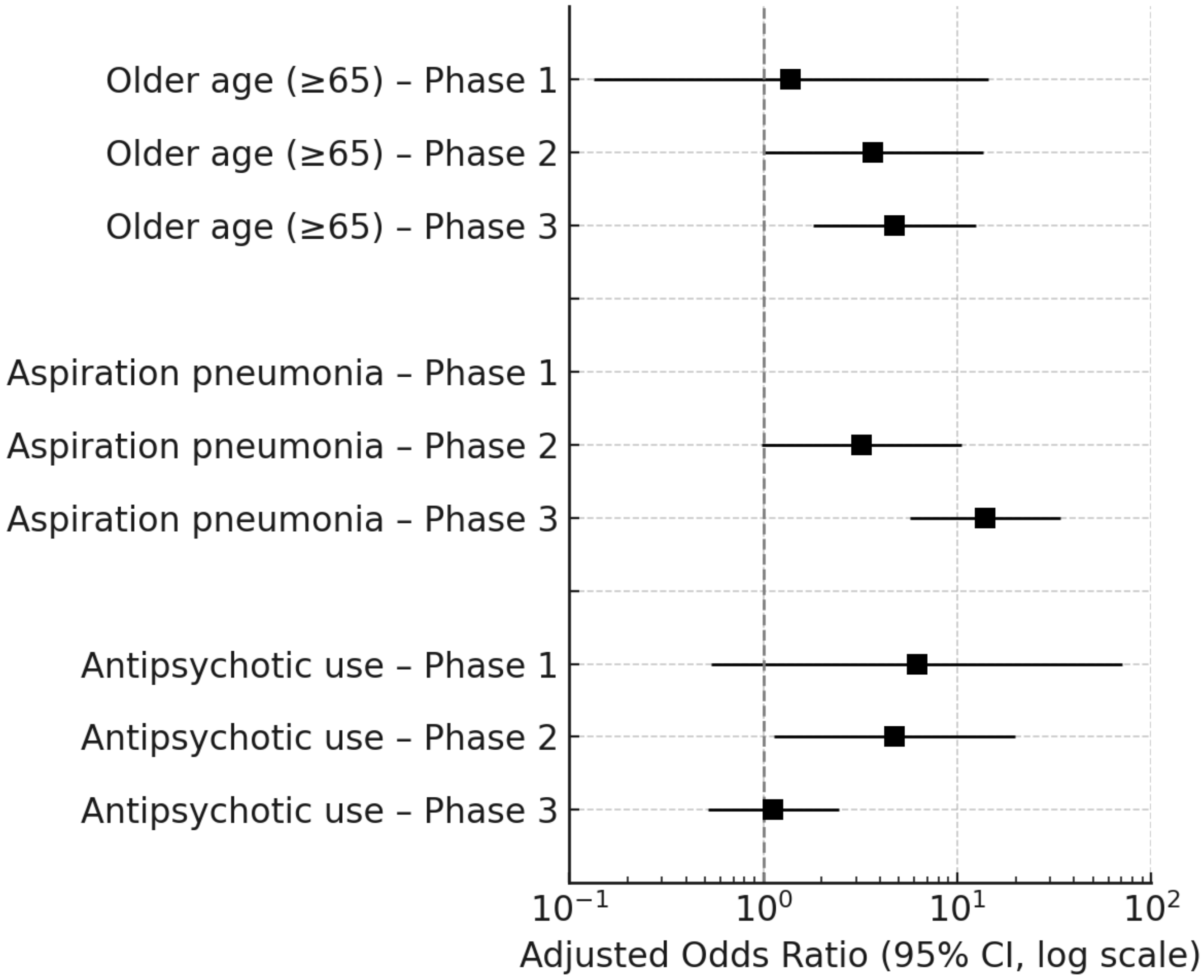

Forest plot of adjusted odds ratios (Model 3) with 95% confidence intervals for three risk factors – older age (≥65 years), aspiration pneumonia, and antipsychotic use – across three pandemic phases (Phase 1: Jun–Dec 2020; Phase 2: Jan–Dec 2021; Phase 3: Jan 2022–Mar 2023). The horizontal axis is on a logarithmic scale.

Supplementary Figure 2. COVID-19 Ward Inpatient Trends with Duration from onset to hospitalization

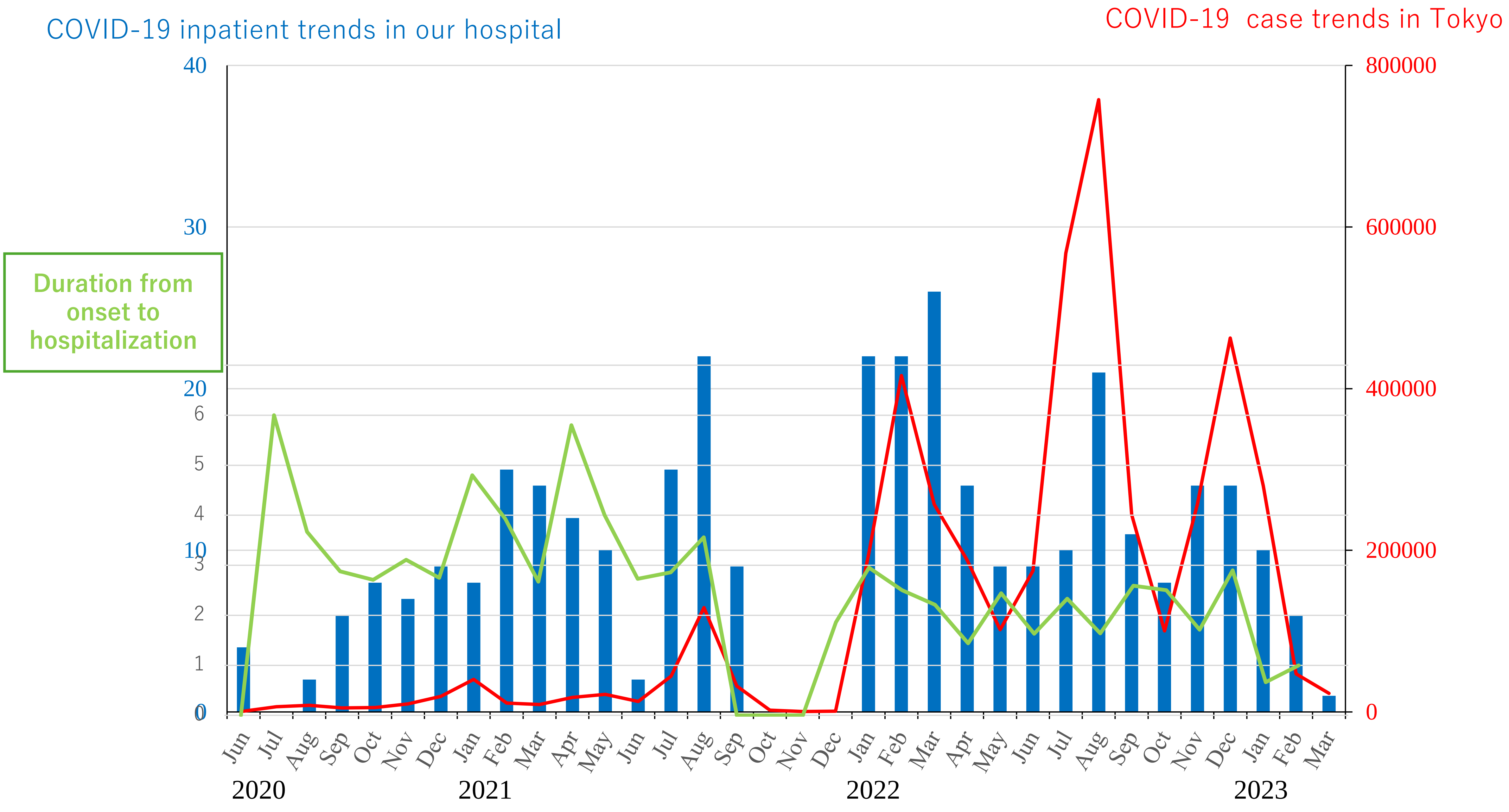

Supplement: Supplementary file 1 — Supporting Information. [file PCN5-4-e70230-s002.pdf]
